# Piezoresistivity and AC Impedance Spectroscopy of Cement-Based Sensors: Basic Concepts, Interpretation, and Perspective

**DOI:** 10.3390/ma16020768

**Published:** 2023-01-12

**Authors:** Amir A. E. Elseady, Ivan Lee, Yan Zhuge, Xing Ma, Christopher W. K. Chow, Nima Gorjian

**Affiliations:** 1 Sustainable Infrastructure and Resource Management (SIRM), UniSA STEM, University of South Australia, Adelaide, SA 5095, Australia; 2 South Australian Water Corporation, Adelaide, SA 5095, Australia

**Keywords:** piezoresistivity, self-sensing concrete, cement-based sensors, AC impedance spectroscopy (ACIS), equivalent circuit model, structural health monitoring

## Abstract

Cement-based sensors include conductive fillers to achieve a sensing capability based on the piezoresistivity phenomenon, in which the electrical resistivity changes with strain. The microstructural characterisation of cement-based sensors can be obtained using a promising non-destructive technique, such as AC impedance spectroscopy (ACIS), which has been recently used by many researchers. This paper reviews the fundamental concepts of piezoresistivity and ACIS in addition to the comparison of equivalent circuit models of cement-based sensors found in the literature. These concepts include piezoresistivity theory, factors affecting piezoresistivity measurement, resistance measurement methodology, strain/damage sensing, causes of piezoresistivity, theories of conduction, AC impedance spectroscopy theory, and the equivalent circuit model. This review aims to provide a comprehensive guide for researchers and practitioners interested in exploring and applying different techniques to self-sensing concrete.

## 1. Introduction

**Figure 1:**
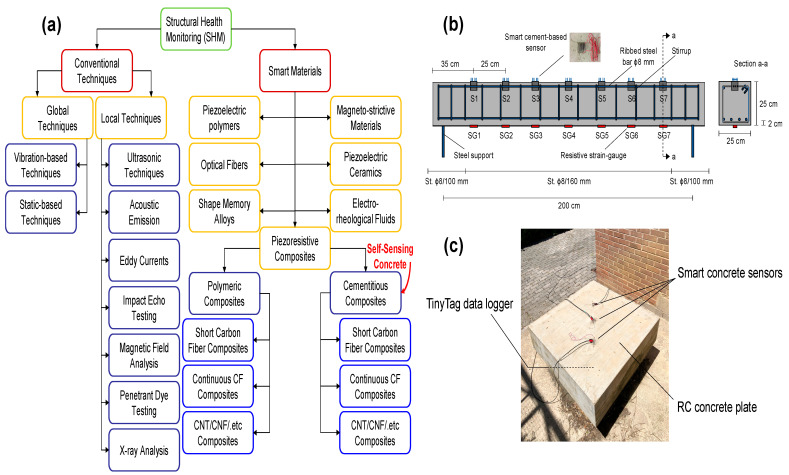
(**a**) Structural health monitoring (SHM) directions; (**b**) Cement-based sensors embedded in RC beams; (**c**) Cement-based sensors embedded in RC plates [13].

The measurement of a structure’s operating environment and signs of deterioration affecting its functioning, serviceability, and safety is generally referred to as “structural health monitoring” (SHM). It requires ongoing monitoring and technical data collection, validation, and analysis to support life-cycle management choices [1]. SHM can be divided into two main directions: traditional techniques and smart materials (Figure 1a), which include cementitious composites that are referred to as “self-sensing concrete” (cement-based sensors), as shown in Figure 1b,c. SHM approaches have been investigated using traditional or more advanced methods that depend on embedded or surface-mounted strain sensors. Fibre Bragg grating is an example of an advanced localised technique, whereas time domain reflectometry in fibre optics is an example of a modern distributed technique of SHM [2,3,4,5,6,7,8]. Previous investigators [9,10] have tried to insert optical fibres into the textile-reinforced concrete (TRC) elements. This sensing system is commonly implemented in structural elements, which necessitates physical adaptation in the load-bearing elements, and as a result, the structural performance may be affected. However, the sensory system is more expensive and more complicated, as it requires special workers. On the other hand, more advanced technologies such as piezoelectric sensors, electrochemical sensors, wireless sensing, and self-sensing concrete are of great importance to researchers [11]. “Self-sensing concrete” refers to the ability of the concrete material to detect its internal stresses, strains, and damage under different loading and environmental conditions without the need for any internal or external sensors [12].

Generally, there are two conditions for attaining self-sensing concrete. The first is by adding conductive admixtures such as carbon-based fillers (short carbon fibres (CF), carbon black (CB), carbon nanotubes (CNT), graphene, etc.) [14]; the second is by conducting current, either alternating current (AC) or direct current (DC) [15], using electrodes. As a result, two approaches can be adopted: resistance-based self-sensing, which is based on resistivity measurements, and capacitance-based self-sensing, which is based on permittivity measurements. The self-sensing approach based on resistance measurement has been frequently used in cement-based materials incorporating various electrically conductive fillers, such as short carbon fibres [16,17,18]. Resistance-based self-sensing is applicable in both low- and high-stress regimes. On the other hand, capacitance-based self-sensing works well only under a low-stress regime [19].

Self-sensing concrete has been used in a variety of investigations to assess its performance, including traffic monitoring [20], corrosion monitoring [21], strain sensing [22], and seismic damage monitoring [23]. Furthermore, numerous investigations have been conducted to determine the properties of self-sensing concrete, including various functional admixtures such as carbon fibres [24], carbon nanofibers [25], carbon nanotubes [26,27,28], graphene nanoplatelets [29], and steel fibres [30].

In 1993, Chung [31] was the first researcher of self-sensing cement-based materials. Following the publication of their report, Chung’s research group [32,33,34,35,36,37,38] and many other research groups [39,40,41,42] released a large number of publications. The self-sensing behaviour of the resistance-based concrete is based on the piezoresistivity theory [43,44], in which the concrete matrix should be conductive under a stimulus like stress or strain. In addition to short carbon fibres, other conductive additives that could be added to the cementitious matrix include carbon black [45,46,47,48], a combination of carbon black and carbon nanotubes [49], carbon nanofibers and carbon nanotubes [50], and steel fibres with micrometre-scale diameters [51]. To describe the degree (sensitivity) of piezoresistivity, a gauge factor (GF) is introduced. The greater the value of the gauge factor, the better the results [19]. It is worth mentioning that GF should be measured within the elastic range to obtain a good indicator of the sensitivity of the conductive fillers used.

The methodology of electrical resistance measurement depends on the type of power source (AC or DC) because of the presence of the polarisation phenomenon [38,52], the configuration of electrodes used (2 or 4-probe) [36,40], and whether the electrodes are embedded in or attached to the specimen [53,54,55]. Both strain (elastic range) and damage (post-elastic range) can be monitored with a resistance-based self-sensing concept. This is because both reversible and irreversible behaviours can be observed for samples under loading [56].

The cause of piezoresistivity in cement-based materials is based on one or more of these factors: the slippage of the fibre–matrix interface [57,58,59,60,61,62], the change in the intrinsic resistance of the conductive admixtures [57,59,63,64], the change in the contact resistance between the functional additives [57,65], the change in the tunnelling distance between the conductive admixtures [57], and the change in the capacitance distance of the conductive fillers [57,66].

The conduction mechanism within cement-based materials is based on one or more of these conduction phenomena: the motion of ions (ionic conduction), which is an inherent part of the water used in the matrix, and the motion of free electrons (electronic and/or hole conduction), which is an inherent part of the conductive fillers added to the cement-based materials to improve their electrical capability [67]. The latter can be divided into contacting conduction, internal field emission conduction, and/or tunnelling conduction (quantum tunnelling) [57]. Understanding the conduction mechanism aims to identify the precise dosage of conductive fillers to attain the percolation threshold at which the conductivity of cement-based materials is acceptable for achieving self-sensing behaviour under loading conditions [57,68]. Moreover, there are many factors affecting the piezoresistivity measurement, such as the filler type, aspect ratio, and dosage [69]; the dispersion of conductive admixtures [70,71]; the type of cement-based matrix [16]; the water-to-cement ratio [72]; the loading type and its amplitude [73], and the ambient environment [16], which includes temperature, relative humidity, and freeze–thaw cycles.

Understanding the microstructural behaviour of the cement-based material is of paramount importance, especially when adding new materials to the concrete matrix to improve its mechanical performance or electrical capability. This enhances the durability and mechanical properties of cementitious materials [74]. Therefore, researchers are trying to use destructive or non-destructive methods. Among non-destructive techniques, alternating current impedance spectroscopy (ACIS) can be used as a real-time non-destructive technique, and it is preferable to the other non-destructive methods [75].

Previously published review papers focused on different aspects of self-sensing concrete and the ACIS technique. For example, Taheri [11] focused on the fabrication of five key sensors, including self-sensing technology; Tian [12] reviewed the materials and fabrication of self-sensing concrete; Baoguo [14] discussed potential structural applications of self-sensing concrete; Dong [16] concentrated on the piezoresistive properties of self-sensing concrete; Abedi [53] focused on the potential application of self-sensing concrete in transport infrastructure; Han [54] reviewed the effect of different carbon-based conductive fillers on self-sensing concrete; Wang [75] discussed the measurement limitations of the ACIS technique; and Hu [76] concentrated on the equivalent circuit models for different cement-based materials. Therefore, for new researchers and practitioners, a comprehensive guide, including the basic principles, is required before going through the previously published studies. This paper provides a comprehensive overview of the topics and the significance of using AC impedance spectroscopy as a non-destructive technique to study the microstructure of self-sensing concrete.

This paper aims to provide a comprehensive guide to both piezoresistivity and ACIS theories proposed in the literature in an insightful way while paying more attention to a deep understanding of their roles. Moreover, the use of AC impedance spectroscopy as a non-destructive technique to study the microstructure of self-sensing concrete is summarised. The paper is divided into two main sections: resistance-based self-sensing concrete and the equivalent circuit model. Section 2 explains the basics of self-sensing concrete, including piezoresistivity theory, factors affecting piezoresistivity measurement, resistance measurement methodology, strain/damage sensing, and conduction theories. On the other hand, Section 3 covers the fundamental concepts of alternating current impedance spectroscopy (ACIS) theory and its equivalent circuit model in light of cement-based materials and sensors. Finally, in addition to the future aspect, a conclusion is drawn.

## 2. Resistance-Based Self-Sensing Concrete

This section will review piezoresistivity theory, factors affecting piezoresistivity measurement, resistance measurement methodology, strain/damage sensing, and conduction theories.

### 2.1. Piezoresistivity Theory

**Figure 2:**
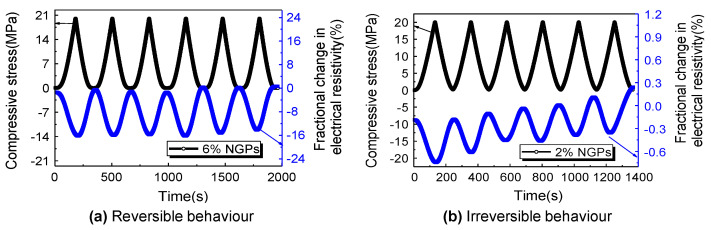
The difference between reversible and irreversible behaviours of electrical resistivity under compressive loading: (**a**) reversible behaviour; (**b**) irreversible behaviour [73].

Self-sensing behaviour is basically attained with piezoresistivity, which is an electromechanical phenomenon in which a material’s electrical resistivity varies with strain in a reversible manner [43], as illustrated in Figure 2. To attain reversibility, the material should be in the elastic range while the piezoresistivity is studied [43]. On the other hand, irreversible behaviour refers to the occurrence of damage in concrete structures [43]. Piezoresistivity is used for strain sensing in scientific measurement. It does, however, provide stress sensing due to the relationship between strain and stress.

The relationship between the volume resistivity and the volume resistance is calculated based on Equation (1).

(1) $$R=\frac{\rho \;l}{A}$$

where *R* represents the resistance of the specimen (volume resistance), ρ represents the resistivity of the specimen (volume resistivity), l represents the distance between voltage terminals, and A represents the cross-sectional area of contact between the electrode and the specimen [43].

Both geometry and resistivity variations affect the change in resistance caused by the applied loads. Note that the resistivity and strain may not be in the same direction [43]. For isotropic material in both directions, the relationship between the fractional change in resistance *(*ΔR/R*)*, resistivity, and strain is defined based on Equation (2).

(2) $$\frac{\Delta R}{R}=\frac{\Delta \rho }{\rho }+Ɛ(1+2\;ʋ)$$

where (Δρ/ρ) is the fractional change in resistivity, *Ɛ* (=Δl/l) is the strain, *ʋ* is the Poisson’s ratio, and (1 + 2 *ʋ*) is the geometric effect [44].

As the change in resistivity (Δρ/ρ) is normally far greater than the change in the strain (Δl/l) in concrete samples [43], it can be assumed that (ΔR/R) equals (Δρ/ρ). Therefore, many investigators reported the relationship between (ΔR/R) and strain (*Ɛ*) in cement-based materials as an indication of piezoresistivity rather than (Δρ/ρ).

The effectiveness of sensing can be represented by the gauge factor (*GF*), as illustrated in Equation (3).

(3) $$GF=\frac{\Delta R/R}{\Delta }$$

The fractional change in resistance per unit strain represents the gauge factor (*GF*). It describes the degree of sensitivity of piezoresistivity. Moreover, its sign depends on the direction of applied loads: positive for uniaxial tension and negative in the case of uniaxial compression. Even if the resistivity does not vary with the applied strain, the resistance does because of the altered dimensions. The gauge factor is nearly two when the resistivity does not change with the applied loads, as the exact number depends on Poisson’s ratio. Conversely, the gauge factor can easily reach two and can exceed it when the resistivity changes with strain [19].

As piezoresistivity is an electromechanical phenomenon in which the material’s resistivity alters reversibly under the applied strain, the gauge factor must be determined in the case of reversible alterations in resistivity due to the applied loads. It should be calculated within the elastic range, as the plastic deformations do not reflect the genuine gauge factor. Additionally, it is preferable to measure the resistance under progressively rising strain amplitudes rather than static loading up to the failure point, as the former state gives a strong indication of the reversibility after unloading [19].

### 2.2. Factors Affecting Piezoresistivity Measurement in Self-Sensing Concrete

It is worth mentioning that there is a difference between resistivity and piezoresistivity. The term “resistivity” describes the electrical resistance status of the cement-based material without loading. Piezoresistivity, on the other hand, refers to the change in the electrical resistance status of the cement-based material in response to a stimulus, whether stress or strain. In other words, piezoresistivity correlates external loads with the resistivity change in the cementitious matrix. Therefore, if the resistivity is affected by any factor, the piezoresistivity will also be affected.

As summarised in Table 1, many factors may affect the sensitivity of piezoresistivity and/or resistivity. To achieve reliable results from the self-sensing concrete, it is advisable to consider as many factors as possible.

### 2.3. Resistance Measurement Methodology

**Figure 3:**
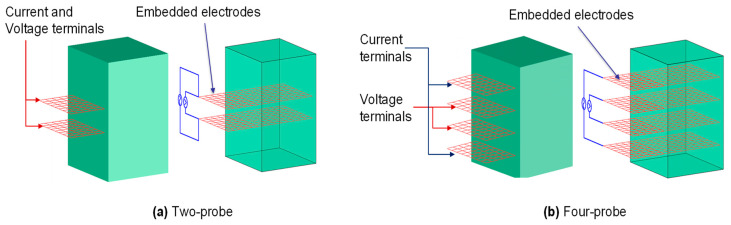
Different electrode configurations for resistance measurement in the cementitious matrix: (**a**) two-probe technique; (**b**) four-probe technique.

The four-probe method is far more dependable for measuring the volume resistance than the two-probe method [36]. Four electrical connections are utilised in the four-probe approach, with the outer two for conducting current and the inner two for measuring the electrical potential difference (voltage) [40], as shown in Figure 3. On the other hand, in the two-probe approach, two electrical connections are utilised, with each probe used for both conducting current and measuring the voltage. Because the contact resistance is removed from the measured resistance, the four-probe method is preferable [36]. Moreover, during the application of strain, the contact resistance can be altered; therefore, a better sensing solution can be achieved by using a grid of contacts, with the exterior for conducting current and the interior for monitoring voltage [112]. Additionally, a two-dimensional or three-dimensional grid of connections can achieve resistivity tomography [113].

Electrodes can be attached to or embedded in the sample. The latter case is preferable to the former one because the apparent or measured resistance will be more accurate and reflect the actual behaviour of the concrete specimen. This is because of the higher contact between the conductive admixtures and the electrodes compared to the former case [53,54,55].

The power source can be DC or AC. The DC approach benefits from a higher degree of current penetration, whereas the AC method benefits from less electric polarisation. Polarisation is the formation of an electric dipole by the mobility of charges (e.g., ions). Polarisation happens during resistance measurement, which requires the measurement meter to provide a small current for the measurement period. The longer the measuring time, the greater the polarisation. When the current polarity is reversed, depolarisation occurs. Because the dipole obstructs conduction, the measured (apparent) resistance is higher than the true resistance. The measurement of electrical resistance within the first few seconds before the polarisation becomes noticeable reveals the true resistance. Another method is to measure the average resistance immediately before and after the polarity reversal [38,52]. Note that the contact resistance should be excluded from the resistance measurement.

**Figure 4:**
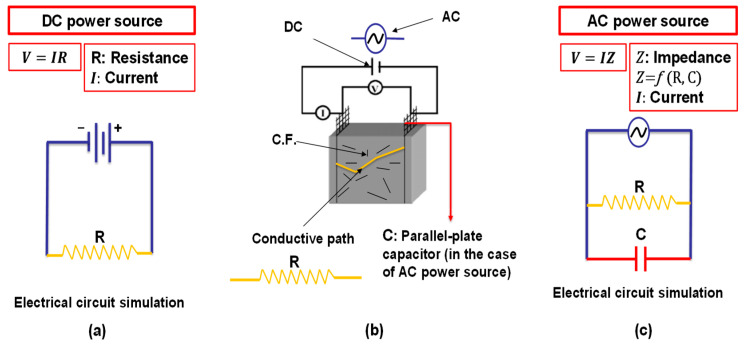
The effect of DC and AC on the electrical resistance measurement of the cement-based sensors: (**a**) DC power source; (**b**) conductive path simulation; (**c**) AC power source.

The DC resistance is distinct from the AC impedance, as the latter includes more complicated measurements such as inductance and capacitance along with the resistance, as shown in Figure 4. Using an AC power source with a high frequency eliminates the effect of capacitance caused by the presence of the two parallel electrodes [81], as shown in Figure 4c.

### 2.4. Strain and Damage Sensing

The electrical resistivity behaviour is reversible in the case of elastic strain, whereas the behaviour is irreversible in the case of fracture [25,56]. This is the idea behind the sensing concept using electrical resistivity. The alteration in electrical resistivity under dynamic tensile stress at the same amplitude is studied by Chung [114]. During the first cycle, minor cracks occurred at the beginning of loading; therefore, an irreversible rise in the resistivity was noticed. Nevertheless, in the subsequent loading cycles, the damage was not observed, and hence the irreversible rise in the resistivity was not spotted [115]. This highlights the precision of the sensing concept, as it detects even minor cracks. On the other hand, fatigue damage is also reported by Chung [116] in cement mortar that contains short carbon fibres as a conductive filler.

**Figure 5:**
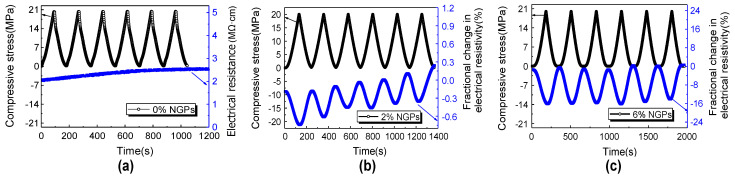
Effect of NGPs dosage on the fractional change in resistivity under compressive loading: (**a**) without NGPs; (**b**) with 2% NGPs; (**c**) with 6% NGPs [73].

The strain-sensing capability is less adequate in the elimination of a conductive admixture, as evidenced by Sun’s [73] use of nanographite platelets (NGPs) in the cementitious matrix with different dosages, as shown in Figure 5, and the reduced signal-to-noise ratio reported by Konkanov [117], using non-conductive additives. Without conductive admixtures, conductivity is dominated by ions rather than electrons, and humidity has a significant impact on the resistance. Electrical conduction, on the other hand, is dominated by electronic conduction with the addition of conductive admixtures at a volume percentage near the percolation threshold (see Section 2.5 for more information), significantly lowering the humidity dependence [67]. Although the humidity reliance is not entirely removed [58], the influence of humidity on the sensing performance is minimal [77].

**Figure 6:**
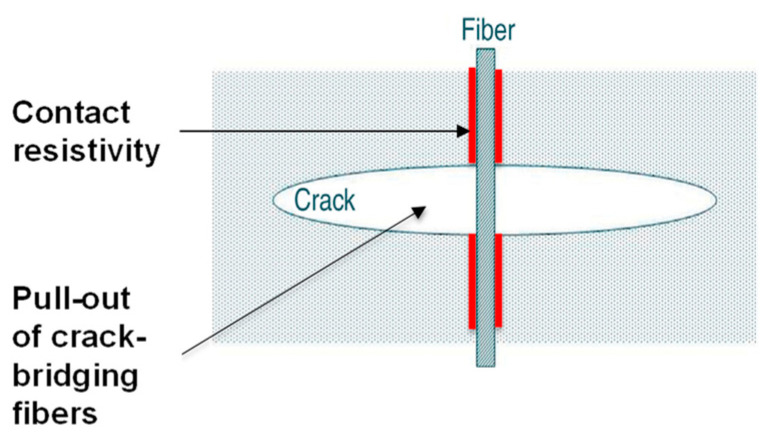
The cause of piezoresistivity in a cement-based matrix containing short carbon fibres [58,116].

Piezoresistivity in a cement-based matrix containing short carbon fibres is caused by a minor slippage of the fibre–matrix interface, as illustrated in Figure 6 [58,116], and an accompanying increase in the interface resistivity [118,119]. There are other causes of piezoresistivity in cement-based materials containing conductive fillers, such as the change in intrinsic resistance of the conductive admixtures, the change in contact resistance between the functional additives, the change in tunnelling distance between the conductive admixtures, and the change in capacitance distance of the conductive fillers [57]. All the previous causes are summarised in Table 2. It is worth mentioning that one or more reasons contribute to the change in piezoresistivity [57].

### 2.5. Conduction Theories

There are two main methods of conduction in cement-based materials. The first method is the motion of ions (ionic conduction), which is an inherent part of the water used in the matrix. The second method is the motion of free electrons (electronic and/or hole conduction), which is an inherent part of the conductive fillers added to cement-based materials to improve their electrical capability. Furthermore, electronic and/or hole conduction includes contacting conduction, internal field emission conduction, and/or tunnelling conduction (quantum tunnelling) [57].

Contacting conduction results from the proximity of functional fillers, which creates a conductive relationship. It is connected to the movement of electrons and/or holes along the conductive channels made by in-contact conductive fillers [57,120]. When electrons in a cement-based material cross the energy barriers (insulating zones between conductive admixtures), tunnelling conduction occurs [57]. The transmission of electrons between the dispersed but sufficiently close-by fillers is related to tunnelling conduction and field emission conduction. It is believed that quantum tunnelling widely occurs in cement-based materials with different conductive admixtures, as it requires a low electric field between conductive admixtures compared to field emission conduction [57,121,122]. However, some functional fillers with distinct morphologies might cause a localised rise in the electric field at sharp ends, thus reducing the width of the barrier and permitting field emission conduction [57,63,123,124]. Previous investigators theorised that quantum tunnelling and field emission conduction were responsible for the conductive behaviour of self-sensing composites [63,83,121,123]. While some investigators may consider internal field emission conduction to be different from tunnelling conduction [57,121,125,126,127], other researchers may consider tunnelling conduction a particular case of field emission conduction [128,129].

Concerning ionic conduction, in addition to calcium silicate hydrate (C-S-H) gel and other solid phases, hydrated cement paste also includes a variety of voids. Ionic species from the solid phases can be dissolved by the water filling these spaces or pores, which causes some ionic conduction through the network of capillary pores. Ionic conductivity varies greatly when cement contains a significant amount of free water because ionic conduction is linked to the migration of ions in the pore solution. The cement matrix resembles an insulating substance as long as it is dried [57,130]. On the other hand, if the concentration of fillers (conductive admixtures) is below the percolation threshold, ionic conduction will dominate in the case of cement-based materials [57,67].

While cement-based materials can barely conduct electrical current based on ionic conduction, they cannot achieve the piezoresistivity (as discussed in Section 2.1) required for them to be used as cement-based sensors. Therefore, conductive fillers should be added to the cement-based matrix. However, not all the dosages of the conductive fillers are sufficient to induce self-sensing behaviour under a stimulus like stress or strain [57,68]. From this point, the percolation phenomenon is introduced to define the optimum dosage of conductive fillers.

**Figure 7:**
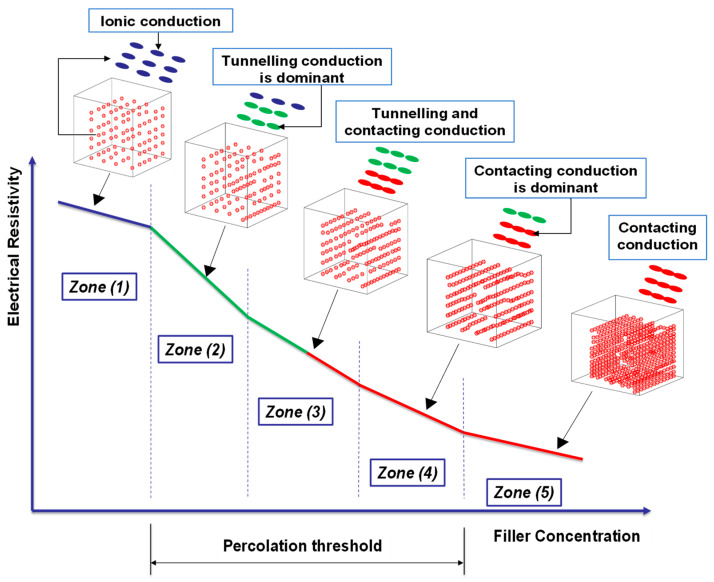
The relationship between the filler dosage and the electrical resistivity without externally applied loads.

The resistivity of the testing material can vary by many orders of magnitude when conductive particles, usually made of metal or carbon, are introduced to a non-conducting matrix. The results obtained by previous investigators indicate that once the particle concentration is raised above a particular threshold level, resistivity abruptly decreases. At that concentration, the system begins to consist of limitless chains of particles. The percentage of particles in the infinite conductive paths increases as the particle concentration rises, thereby helping with the conduction process [131,132,133]. Figure 7 (zones 2, 3, and 4) shows that increasing the dosage of conductive fillers results in a precipitous decrease in resistivity, and this range is called the percolation threshold. The volume fraction at which the conductive fillers contact each other (contacting conduction) is called the percolation threshold [134]. However, contacting conduction is not the only way to attain the percolation threshold. If the conductive fillers are close enough to cross the energy barriers (tunnelling conduction and/or field emission conduction), the percolation threshold can also be achieved [135]. There is no identified dosage of conductive fillers to attain the percolation threshold, as it depends on many variables such as the composite’s microstructure, the type of conductive fillers, their aspect ratio, their orientation, and the presence of externally applied loads [134,136].

Based on the previous discussion, it can be inferred that the percolation threshold depends not only on the contacting conduction but also on the quantum tunnelling and field emission conduction. In other words, the percolation threshold describes the conduction at the macro level, and both tunnelling and field emission conduction describe the conduction at the micro level [137]. Figure 7 shows the relationship between the dosage of conductive fillers and the corresponding resistivity of an arbitrary cement-based matrix without externally applied loads. Zone 1 reveals the domination of ionic conduction because the dosage of fillers is very low. Zone 5, on the other hand, exhibits the control of contacting conduction due to the high dosage of conductive fillers. In the meantime, the percolation range (zones 2, 3, and 4) represents the alterations in conduction type (contacting and tunnelling) with increasing the filler dosage, and this conduction mechanism is comparable to Hui’s [83] results. If the fibre content is around the percolation threshold, the electrical resistivity will be low, and the piezoresistivity will be strong; hence, the resistivity alters significantly with strain. In the case of using fibre content above the percolation threshold, the overall cost and sensing effectiveness may be affected [138,139,140].

**Figure 8:**
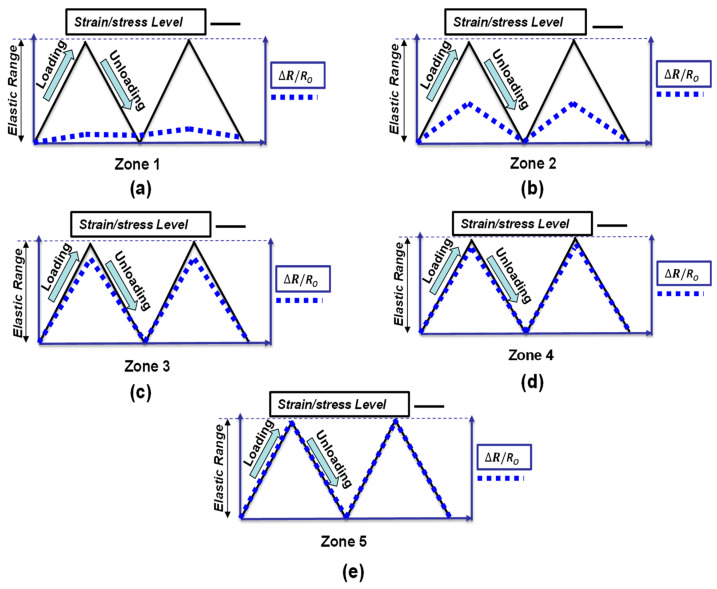
The relationship between the fractional change in resistance and filler concentration under externally compressive loading: (**a**) zone 1; (**b**) zone 2; (**c**) zone 3; (**d**) zone 4; (**e**) zone 5.

The relationship between the fractional change in resistance (ΔR/R) and the externally applied loads (strain or stress) within the elastic range is illustrated in Figure 8. It can be noticed that increasing the dosage of fillers causes the pattern of fractional change in resistance to coincide with the loading pattern. This is because of the effect of conductive fillers that increase the number of conductive passages within the cement-based matrix. Figure 8 can be correlated to Figure 7, depending on the pattern of fractional change in resistance. For example, Figure 8a shows that the fractional change in the resistance pattern is irreversible under loading; this can be correlated to zone 1 in Figure 7, which shows the ionic conduction dominance. It is worth mentioning that the position and orientation of conductive fillers change under loading, and this alteration affects the reversibility pattern of the fractional change in resistance depending on the loading type (tension or compression) [141].

## 3. Equivalent Circuit Model

AC impedance spectroscopy has been suggested as a promising, non-destructive method for examining self-sensing concrete. In other words, this method can be used to study the microstructure composition of self-sensing concrete in terms of fibre orientation, fibre dosage, etc. This section will discuss the fundamentals of AC Impedance Spectroscopy (ACIS) in light of cement-based materials. Additionally, the equivalent circuit models of different cement-based sensors will be summarised.

### 3.1. ACIS Theory

A more generalised version of electrical resistance is impedance. Impedance includes the main components of electrical resistance, such as resistors, capacitors, and inductors, as illustrated in Table 3. Both direct current (DC) and alternating current (AC) can be used as an excitation voltage. However, it is preferable to use AC because it is more sensitive and covers a wide range of chemical reactions inside samples by using small perturbation signals compared to DC, which gives responses at a relatively large perturbation depending on the composition of the testing materials [142,143]. Impedance spectroscopy (IS) can be split into two major types depending on the frequency range. In the frequency domain ranging from sub-m Hz to k Hz, the electrochemical reactions between electrodes and liquids in batteries can be studied; in this case, such impedance spectroscopy can be called electrochemical impedance spectroscopy (EIS). On the other hand, ACIS can be utilised when the frequency domain ranges from Hz to MHz to study the solid-solid interface or the solid-liquid interface in cement-based materials [144].

**Figure 9:**
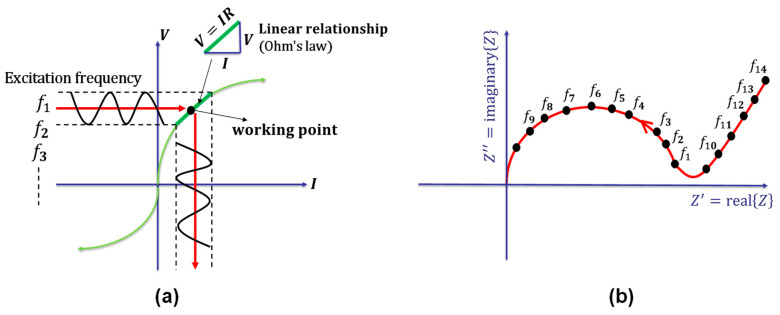
Fundamentals of impedance spectroscopy: (**a**) the voltage-current relationship; (**b**) a complex plane (the Nyquist plot) [148].

ACIS can be used to study the microstructural behaviour of the cement-based material under an external voltage perturbation. The ratio between the applied voltage and the output current represents the total impedance, reflecting the internal properties of the cement-based material [143]. Single or multi-sine waves with different frequencies and phases can be applied simultaneously in the time domain. Then, using a Fourier transform (FT), the current in the frequency domain can be resolved [142,145]. It is worth mentioning that the applied perturbation should not be very high to ensure the linearity between the voltage and current, as shown in Figure 9a [142,145,148]. Applying a wide frequency range detects different physical and chemical phenomena within the testing sample [148].

**Figure 10:**
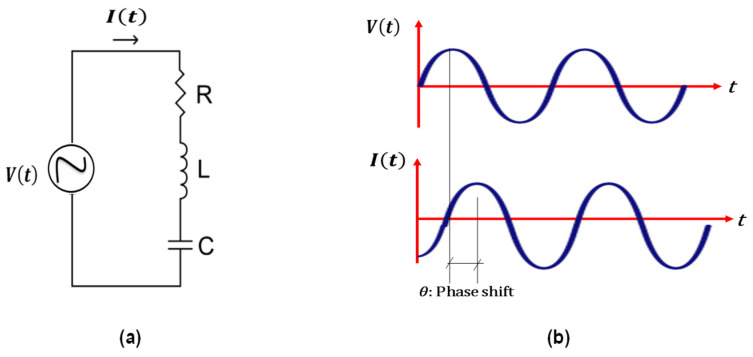
(**a**) a simple AC circuit (RLC) with a resistor, a capacitor, and an inductor in series; (**b**) the phase shift (𝜃 = 0 for a resistor; 𝜃 = +90° for a capacitor; 𝜃 = −90° for an inductor).

ACIS can be used to analyse a simple AC circuit with a resistor, a capacitor, and an inductor in series, as shown in Figure 10a, in the time domain with a voltage perturbation (v(t)= $v_{0}$ sin(wt)).

The relationship between the different quantities and the applied perturbation in the time domain can be deduced as illustrated in Equation (4) [143,145].

(4) $$L\frac{dI}{dt}+RI+C^{-1}\int Idt=V\left(t\right)$$

where I is the current, *R* is the resistance, *C* is the capacitance, and *L* is the inductance.

Using the Fourier transform to change Equation (4) from the time domain to the frequency domain, as shown in Equation (5) [143,145].

(5) $$\left(i\omega L+R+{\left(i\omega C\right)}^{-1}\right)\tilde{I}=\tilde{V}\left(\omega \right)$$

where ω is the angular frequency that equals 2πƒ (ƒ is the frequency of the AC source), i is the square root of (−1).

It can be inferred from Equation (5) that the total impedance, as illustrated in Equation (6), depends on the frequency domain rather than the time domain [143,145].

(6) $$Z_{t}=\frac{\tilde{V}\left(\omega \right)}{\tilde{I}\left(\omega \right)}$$

For a series circuit of a resistor, a capacitor, and an inductor, the total impedance is calculated as shown in Equation (7) [145].

(7) $$Z_{t}=i\omega L+R+{\left(i\omega C\right)}^{-1}$$

To obtain the effect of a resistor alone, make the other two quantities equal to zero in Equation (7). Following the same pattern, the effects of a capacitor alone, an inductor alone, a series circuit of a capacitor and an inductor, and a parallel circuit of a resistor and a capacitor can be obtained, as shown in Table 3.

Solving Equation (5) for the whole frequency range is very complex from a mathematical point of view and leads to complicated mathematical operations. As a result, it is much easier to solve it using ACIS for a given frequency, ω, with only two variables: the amplitude and the phase shift (illustrated in Figure 10b) [143]. A complex representation (Nyquist plots) in the polar or rectangular form (the rectangular form used in the following explanation, Figure 9b) can be used to display the two quantities as a single frequency. It is worth mentioning that the imaginary part of the complex plane is just for mathematical manipulations, and there is nothing fictitious about the physical quantity impedance [142].

**Figure 11:**
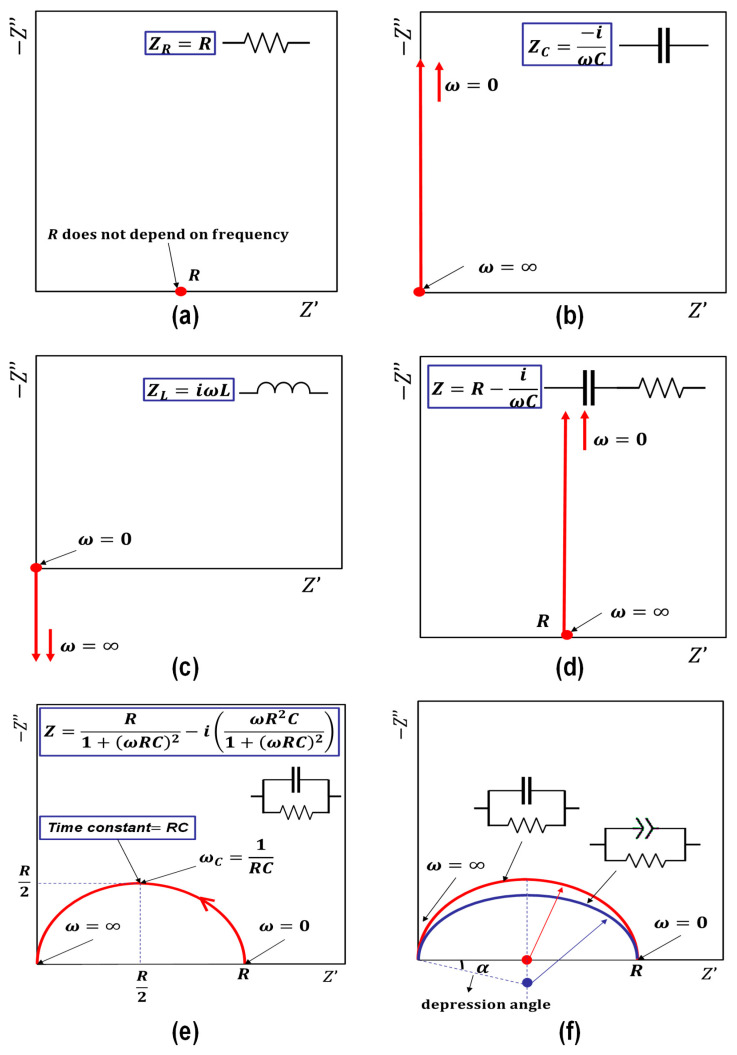
Complex plans (Nyquist plots) of typical ACIS plots for different combinations of a resistor, a capacitor, and an inductor: (**a**) the representation of a resistor; (**b**) the representation of a capacitor; (**c**) the representation of an inductor; (**d**) the representation of a capacitor and a resistor in series; (**e**) the representation of a capacitor and a resistor in parallel; (**f**) the representation of a constant phase element (CPE).

A representation of the typical combinations of a resistor, a capacitor, and an inductor in the complex plane is shown in Figure 11. Substituting ω with (0) and $\left(\infty \right)$ into each equation in Figure 11 gives the graphic representation of each case. Besides substituting ω with (0) and $\left(\infty \right)$, an additional value can be added at ω = (*1*/*RC*) in Figure 11e. This leads to the highest value of ω, which in this situation will be called the characteristic frequency $\omega_{C}$; the inverse of it is called the time constant, which is related to a particular phenomenon within the testing material [143,145]. The semicircle depicted in Figure 11e is the foremost representation of ACIS because it represents the case of a parallel capacitor and resistor, from which the cement-based characteristics can be deduced, as illustrated in the following section. Further reading on the basics of impedance spectroscopy has been interpreted in detail [149].

**Figure 12:**
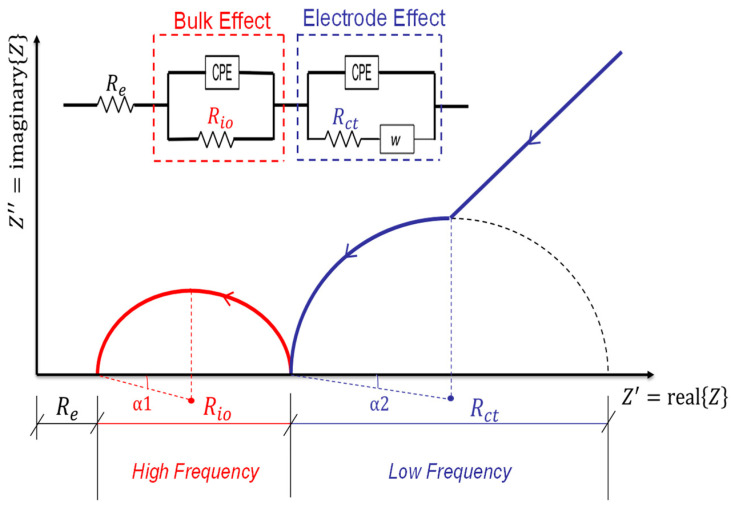
The equivalent circuit model of McCarter [150] and its representation on the complex plane.

Although physical and chemical phenomena may occur at various frequencies, the time constant or the characteristic frequency can discriminate between them. The different phenomena will emerge as distinct arcs, as illustrated in Figure 12, if their time constants vary by at least two orders of magnitude [144]. Otherwise, there may be an overlap between the two arcs, necessitating computational techniques to determine the values of *R* and *C* (*R* is the electrical resistance, as illustrated in Equation (1), and *C* is the capacitance calculated from the following relationship: *C =* k ε *o A/L*, where k is the relative permittivity (dielectric constant), ε*_o_* is the permittivity of free space (8.85 × 10^−12^ F /m), and *A* and *L* are as defined in Equation (1)). As a result, the time constant of a particular material depends more on its resistivity and relative permittivity (dielectric constant) than on its geometry [144]. Since the behaviour of various circuit components may be used to understand many physical processes, the equivalent circuit model, which is covered in greater detail below, can simulate a system’s reaction.

### 3.2. Equivalent Circuit Model and the Corresponding Physical Meaning

**Figure 13:**
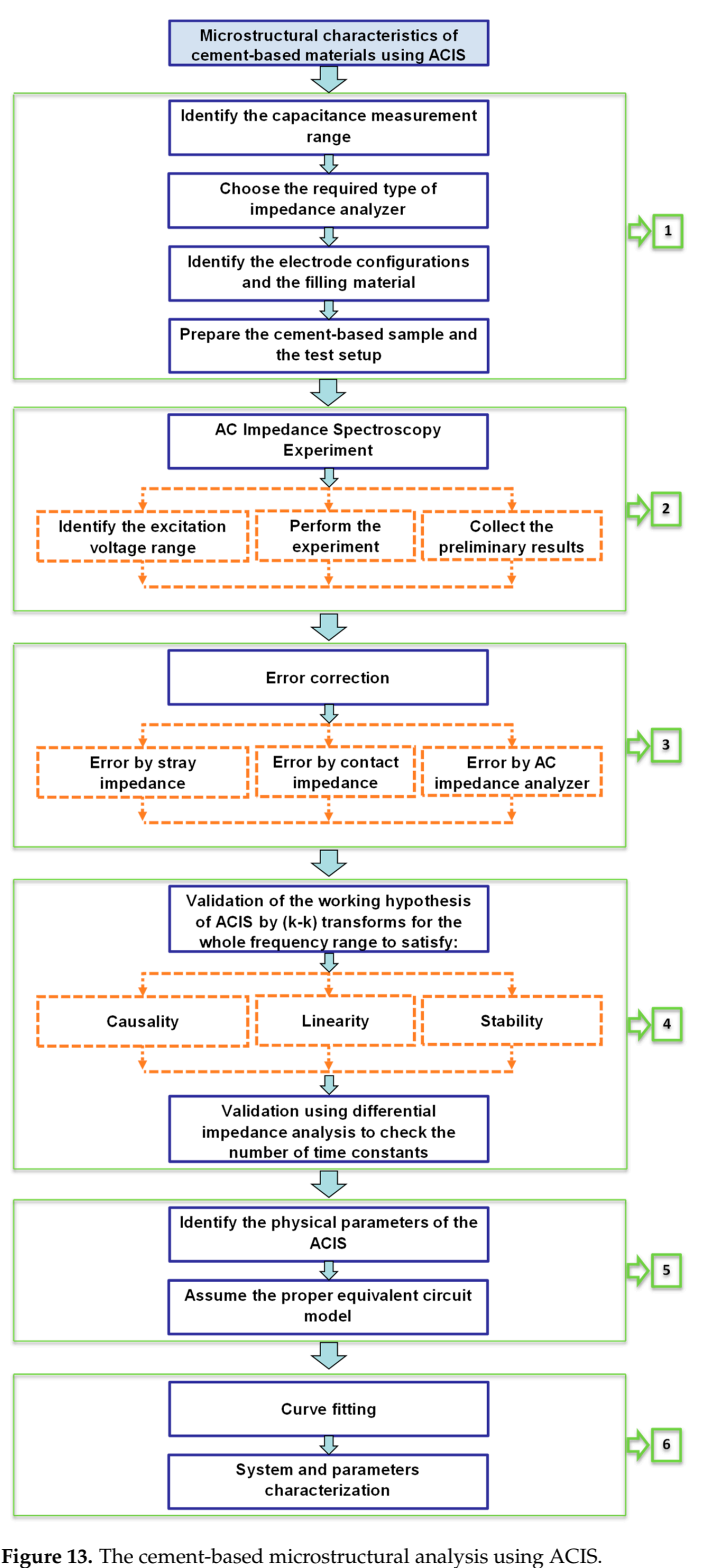
The cement-based microstructural analysis using ACIS.

**Figure 14:**
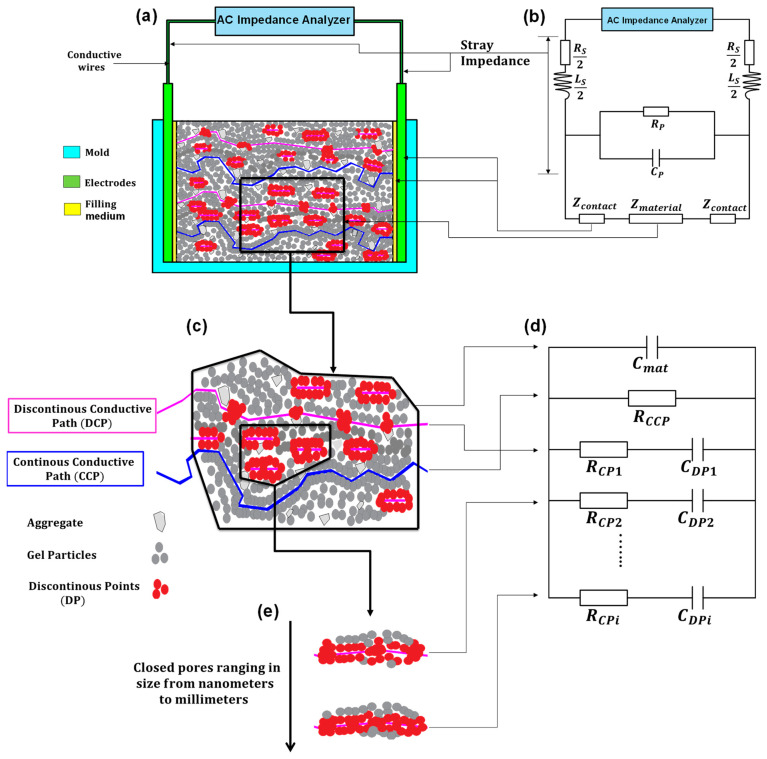
The equivalent circuit model and the corresponding physical meaning in the case of cement-based materials: (**a**) the preparation of a cement-based sample; (**b**) the electrical representation of stray impedance; (**c**) the simulation of DCP and CCP; (**d**) the equivalent circuit model; (**e**) the simulation of closed pores.

The required steps for applying the ACIS technique are illustrated in a flow chart, as shown in Figure 13. The chart consists of six main steps. Step 1 includes preparing samples, selecting the electrode type and configuration, and identifying the AC impedance analyser range. Step 2 contains carrying out the test and collecting the preliminary results. As shown in Figure 14a,b, step 3 represents error correction, which includes stray impedance and contact resistance [75]. Step 4 incorporates the validity of the preliminary results to ensure causality, linearity, and stability, followed by differential impedance analysis to check the number of time constants. In step 5, the physical parameters and the equivalent circuit model can be identified. Finally, step 6 contains the curve fitting and the system characterisation [75,144,151,152,153,154,155].

The concept of the equivalent circuit model needs to be introduced to analyse the resulting ACIS measurement obtained from a particular material. The equivalent circuit model comprises resistors, capacitors, and inductors to simulate the resulting ACIS measurement. In other words, the total impedance of the equivalent circuit model, which contains the three elementary components, should match the resulting ACIS measurement at every frequency [143].

To construct a proper equivalent circuit model, it is advisable to adopt a physical model describing the different parameters (resistors, capacitors, and inductors) of the ACIS measurement. Several models attempted to simulate the bulk and microstructural properties of cement-based materials, including the layer model [156], the brick model [157], the T and I model [158], the barrier/hole model [159], and the conductive path model [74,153,160,161,162,163]. It is worth mentioning that the conductive path model, which represents the best model describing the actual composition of the cement-based materials, can be used to construct the equivalent circuit model [75].

Most of the phenomena (the electrode/sample interface, cement hydration, and the solid–liquid interface) can be represented in Figure 11f by a parallel resistor and capacitor, forming the ideal semicircle (red colour). However, the resulting semicircle of the ACIS measurement is, in most cases, depressed below the real axis by a depression angle (*α*), as shown in Figure 11f (blue colour). This is because the ideal capacitor does not exist in reality, and thus a non-ideal capacitor (a constant phase element, or CPE) is introduced, as illustrated in Table 3 [143,144,150]. The constant phase element, including the depression angle, can be affected by the dispersion of relaxation times and the pore size distribution of cement paste [150,164,165].

**Figure 15:**
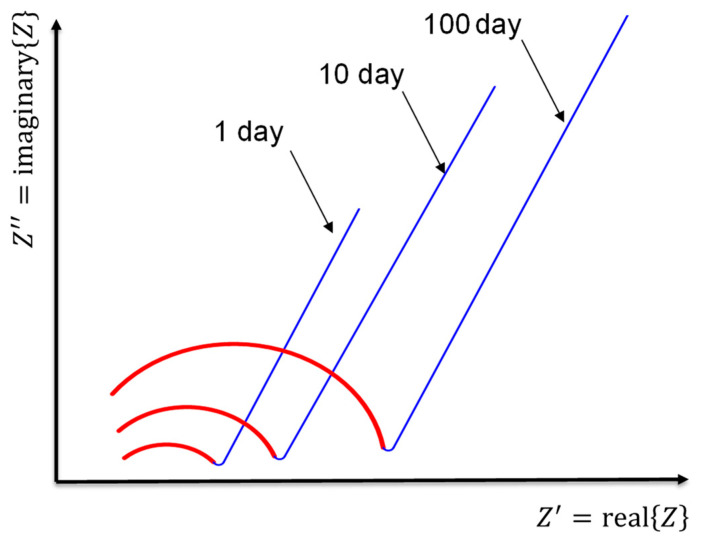
Nyquist plots of the cement-based samples of McCarter [150] over a period of 1, 10, and 100 days.

The equivalent circuit model will simulate the bulk and microstructural properties of the cement-based materials. Under the effect of the electrical field used to carry out the ACIS measurement, the electrical response of most materials that contain different compositions is heterogeneous [144]. This response at least includes both bulk material and electrode/sample responses, as shown in McCarter’s model [150] in Figure 12. In this model, in the low-frequency range, which may be excluded from the ACIS measurement if the cut-off frequency is used [75], the electrode/sample interface is detected in the ACIS measurement. This can be seen in the presence of the blue colour in Figure 12, which contains the Warburg element (W) that represents the diffusion at the electrode/sample interface (see Table 3), the constant phase element (CPE) that represents the spread of relaxation times, and the charge transfer resistance ($R_{ct}$). In the high-frequency region, the bulk response of the cement-based sample appears as the red semicircle shown in Figure 12. This semicircle includes the CPE and the resistance ($R_{io}$) that represents the resistance of ionic conduction. Note that ($R_{e}$) means that the semicircle is not intersected with the origin and does not refer to any physical meaning. McCarter [150] tried to study the properties of the hardened cement-based material for 1, 10, and 100 days. Figure 15 reveals that increasing the degree of hydration leads to a rise in both the capacitance and resistance of the cement-based material. This is just one idea behind studying the microstructure of cement-based materials using the ACIS measurement.

Furthermore, more complicated properties, such as cement hydration and a solid/liquid interface, can be detected, as shown in Figure 14a,c,e. The equivalent circuit model of Song [74] is based on the conductive path model, which contains a continuous conductive path (CCP), a discontinuous conductive path (DCP), and an insulator path (IS). Song considered that the products of the hydration process, like the C-S-H gel, were isolating particles, the unconnected pores were a discontinuous conductive path, and the connected pores were a continuous conductive path, as shown in Figure 14c. Moreover, the continuous conductive path is represented by a resistance ($R_{CCP}$) because of the resistance of the ionic conduction, the discontinuous conductive path with a resistance ($R_{CP}$) as well as a double layer capacitor ($C_{DP}$) that emerged because of the existence of discontinuous points, and the bulk resistance of the sample is represented by a capacitor ($C_{mat}$) because of the presence of the outer electrodes and the dielectric material (cementitious matrix) in between [149], as illustrated in Figure 14d.

Generally, Song’s model is represented by three parallel branches only ($C_{mat}$, $R_{CP}C_{DP}$, and $R_{CCP}$), as shown in Figure 14d. However, it is imperative to remember that cement-based materials commonly have closed pores ranging in size from nanometres to millimetres [152]. Therefore, one discontinuous conductive path is not sufficient to simulate the actual case of the unconnected pores [75,158]. Consequently, the final representation of the cement-based materials can be expressed as shown in Figure 14c–e, which satisfies the Maxwell model that contains an infinite number of parallel branches [147]. The total impedance of the Maxwell model can be calculated through Equation (8).

(8) $$Z_{t}={\left\{R_{ccp}^{-1}+iwC_{mat}+\sum_{k=2}^{k=n}{\left[R_{CPk}+{\left(iwC_{DPk}\right)}^{-1}\right]}^{-1}\right\}}^{-1}$$

where w is the angular frequency, and i is the imaginary number.

It is worth mentioning that many models were developed to characterise the different physical properties of cement-based materials based on the admixtures used, and each model can be interpreted in various ways. Besides studying the effect of the hydration process using ACIS measurement, as has been discussed earlier, other advantages of using the ACIS measurement in the case of cement-based materials include studying the effect of the chloride diffusivity [76,166], the effect of various temperatures and humidity [167], the effect of fly ash in blended OPC mortars [168], the effect of the influencing factors in a novel repairing material [169], the effect of brick powders as a partial replacement for fine aggregates [170], the effect of nanomaterials in cement-based mortars [171], and the effect of mineral admixtures on the durability of prestressed concrete cylinder pipe [172].

### 3.3. Equivalent Circuit Models of Cement-Based Sensors

Based on the previous discussion of ACIS principles, the following different models of cement-based sensors in Table 4 can be interpreted. The presence of conductive fillers, which have many types and shapes, affects both the capacitance and resistance of the equivalent circuit model. As a result, no identified model can be followed, and more research is needed to reach a general model for cement-based sensors.

## 4. Challenges and Future Aspects

Even though self-sensing concrete has been studied for more than three decades, more investigations are required to facilitate its use in real-life applications. This is because the technology depends on many factors, such as the type of conductive filler, the dosage, the aspect ratio, and, most importantly, environmental impacts at the time of sensing. Therefore, it is vital to narrow down the types of conductive filler that will attain the best performance relating to piezoresistivity, behaviour under different environmental conditions, and cost-effective dosage. On the other hand, ACIS is a promising non-destructive technique to study the microstructural compositions of cement-based materials. However, more investigations are required to identify a general equivalent circuit model to simulate cement-based sensors.

## 5. Conclusions

In this paper, the fundamental concepts required for new research in the field of self-sensing concrete have been discussed, as well as how to study the microstructural composition of cement-based materials and sensors using the ACIS technique. A review of the relevant literature draws the following conclusions:

1. Piezoresistivity is a phenomenon that achieves self-sensing in cement-based sensors by distinguishing between reversible and irreversible behaviours. Moreover, the sensitivity of the piezoresistivity can be measured using the gauge factor (GF).
2. Piezoresistivity depends on many factors that affect its reliability.
3. The resistance measurement methodology depends on the properties of AC and DC power sources in addition to the configuration of the electrode. Additionally, using an AC power source with a high frequency is preferable to using a DC power source.
4. In general, the cause of piezoresistivity in cement-based sensors depends on the orientation and displacement of conductive fillers included in the cement-based matrix under loading.
5. The percolation threshold depends on the type of conductive filler, the dosage, and the aspect ratio. It can be attained through contacting conduction and the quantum tunnelling phenomenon.
6. The ACIS theory and the equivalent circuit model can effectively characterise the microstructure of cement-based sensors as a non-destructive technique.

## Figures and Tables

**Table 1:** Factors affecting resistivity and piezoresistivity measurement in self-sensing concrete.

Factor	Description	Key Findings	Refs.
The filler type, the aspect ratio, and the dosage	The type of conductive filler (CF, CNT, CB, etc.), as well as the geometry and dosage, affect the resistivity of cement-based composites.	The change in conductive fillers’ geometry and dosage affects the formation of the conductive passages, leading to an alteration in the percolation threshold.	[69,77,78,79,80,81,82,83,84,85,86,87,88]
The dispersion of conductive fillers	The dispersion of conductive fillers is advisable to form the conductive passages and improve the workability of concrete.	Using supplementary cementitious materials (silica fume, fly ash, and slag) and chemical admixtures (latex, methylcellulose, and superplasticisers) enhances the dispersion of conductive fillers. However, the impact on resistivity and piezoresistivity is different.	[38,67,70,71,77,89,90,91,92,93,94,95,96,97]
The matrix type	The type of cementitious matrix (cement paste, mortar, and concrete) affects resistivity.	In the presence of conductive fillers, cement paste is more conductive than mortar and concrete; fine and coarse aggregates hinder the formation of conductive paths.	[16]
The water-to-cement ratio	The water-to-binder ratio affects the resistivity and piezoresistivity.	The piezoresistivity stability improves when the water-to-cement ratio is reduced. However, this may affect the rheological properties of the cement-based material.	[72,98,99]
The curing type and its duration	The method of curing (moist or air) and its duration affect cement-based materials’ resistivity and piezoresistivity.	Samples tested at 28 days showed better reversibility compared to 7 and 14 days.	[25,100]
The loading type and its amplitude	The loading type’s amplitude and frequency affect the piezoresistivity.	The piezoresistivity is affected differently by monotonic and cyclic, uniaxial, biaxial, and multiaxial forces.	[73,101,102,103,104]
The electrode configuration	The electrode configuration, such as embedded, attached, two contacts, four contacts, electrode material, and electrode position, affect the resistivity and piezoresistivity.	The four-probe technique is more reliable than the two-probe technique; embedded electrodes are better than attached electrodes, and the distance between electrodes does not significantly impact the measurement. Additionally, the resistivity measurement does not depend on the area of the voltage probes.	[36,40,99]
The power supply type (DC or AC)	Current type (AC or DC), intensity, and lasting time affect piezoresistivity.	An AC power source is generally better than a DC power source, and a high frequency is preferable to a low frequency.	[25,99,105]
The freeze-thaw cycles	The damage to cement-based materials due to the freeze-thaw cycles is primarily caused by the freezing of water inside pores. This damage can be quantified using the change in resistivity.	The impact on resistivity due to the freeze-thaw cycles is minimal compared to the temperature impact on resistivity.	[106,107,108,109]
The temperature	The change in external temperature affects the resistivity and piezoresistivity.	Increasing the temperature leads to a decrease in the resistivity of cement-based materials.	[97,99,110]
The relative humidity and the moisture content	The change in relative humidity and moisture content affects the resistivity and piezoresistivity.	At low dosage of conductive fillers, the relative humidity and water content affect the resistivity. Conversely, increasing the dosage of conductive fillers leads to a reduction in this impact. Moreover, the presence of water leads to a longer measurement time as the polarisation is enhanced.	[58,81,111]

**Table 2:** Causes of piezoresistivity in cement-based sensors.

Cause	Description and Key Findings	Refs.
The slippage of the fibre–matrix interface	The pull-out of crack-bridging fibres during crack opening leads to an increase in the contact electrical resistivity.	[57,58,59,60,61,62]
The change in intrinsic resistance of the conductive admixtures	Under the externally applied loads on the concrete matrix, deformations occur in the conductive fillers, leading to changes in their intrinsic resistance.	[57,59,63,64]
The change in contact resistance between the functional additives	Under the externally applied loads on the concrete matrix, the position of the conductive fillers alters, leading to direct contact or separation between them. As a result, an increase or decrease in the contact resistance between conductive fillers occurs.	[57,65]
The change in tunnelling distance between the conductive admixtures	Under the externally applied loads on the concrete matrix, the tunnelling distance, or the insulating distance, of the cementitious layer between the functional fillers alters, leading to an alteration in the electrical resistance of the composite.	[57]
The change in capacitance distance of the conductive fillers	At the microstructural level, carbon fibres may be considered micro-capacitance plates because of the ionic conduction between them in the concrete matrix. Therefore, under the externally applied loads on the concrete matrix, the distance between these micro-capacitance plates alters, leading to a change in the resistance of the composite.	[57,66]

**Table 3:** Common electrical circuit components of AC circuits.

Symbol	Description	Impedance	Variables
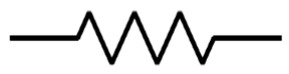	A resistor with resistance, R [Ω, “ohms”]	$Z_{R}=R$	$Z_{R}$ is the impedance due to a pure resistor [145].
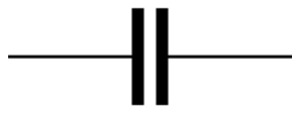	A capacitor with capacitance, C [F, “farads”]	$Z_{C}=\frac{-i}{\omega C}$	$Z_{c}$ is the impedance due to an ideal capacitor, i is the square root of (−1), and ω is the angular frequency and equals 2πƒ, where ƒ represents the frequency of the AC source [145].
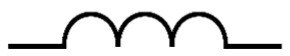	An inductor with inductance, L [H, “henrys”]	$Z_{L}=i\omega L$	$Z_{L}$ is the impedance due to an ideal inductor [145].
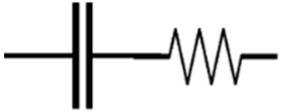	A resistor and a capacitor in series	$Z_{t}=R-\frac{i}{\omega C}$	$Z_{t}$ is the impedance due to a pure resistor and an ideal capacitor in series [145].
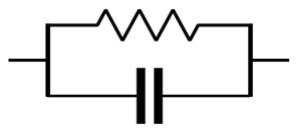	A resistor and a capacitor in parallel	$Z_{t}=\frac{R-i\;\omega R^{2}C}{1+{\left(\omega RC\right)}^{2}}$	$Z_{t}$ is the impedance due to a pure resistor and an ideal capacitor in parallel [145].
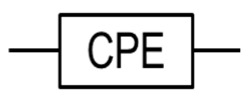 or 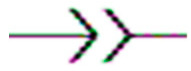	The constant phase element (CPE) can be used to represent the non-ideal behaviour of a capacitor, as there are losses in capacitance due to relaxation time spread and dielectric dispersion.	$Z_{CPE}=\frac{1}{Q_{0}{\left(i\omega \right)}^{\alpha }}$	$Z_{CPE}$ is the impedance due to a complex circuit component, $Q_{0}$ is a pseudo-capacitive coefficient, and α is an arbitrary constant with no physical meaning and its value between 0 and 1 (0 ≤ α ≤ 1). If α = 1 then $Z_{CPE}=Z_{C},\;\mathrm{and}\;\mathrm{If}\;\alpha =0\;\mathrm{then}\;Z_{CPE}=Z_{R}$ [145,146].
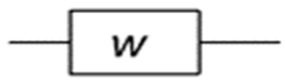	The Warburg element (W) can be used to simulate the semi-infinite diffusion at the electrode/sample interface.	$Z_{w}$ = $\frac{\sigma }{{\left(i\omega \right)}^{1/2}}$	$Z_{w}$ is the impedance of the Warburg element, and 𝜎 is the Warburg parameter [147].

**Table 4:** Summary of equivalent circuit models related to different cement-based sensors.

Equivalent Circuit Model	Parameters	Specification	Limitations	Ref.
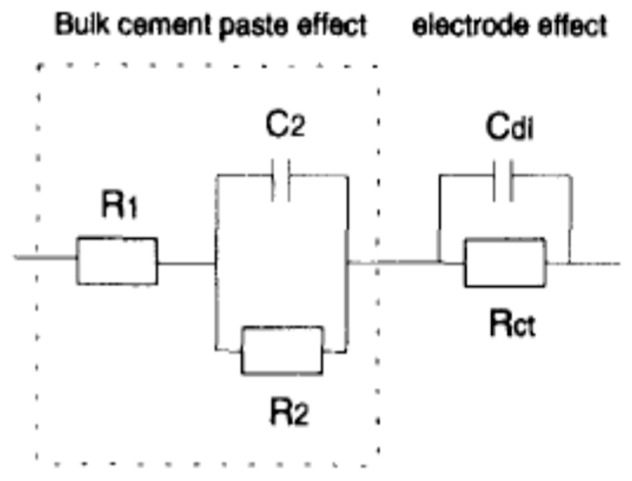	R_1_ is the high-frequency resistance, R_2_ is the resistance of solid/liquid interface, and C_2_ is the bulk capacitance. R_ct_ is the charge transfer resistance of the cement/electrode interface, and C_dl_ is the double-layer capacitance.	The model was used to study the crack growth of cement-based composites reinforced with polypropylene, carbon fibre, and mica flakes under a compressive load.	The model can characterise and detect crack growth in cement-based composites containing polypropylene fibres and mica flakes. However, it did not reflect the microstructure of cement-based materials incorporating carbon fibres.	[173]
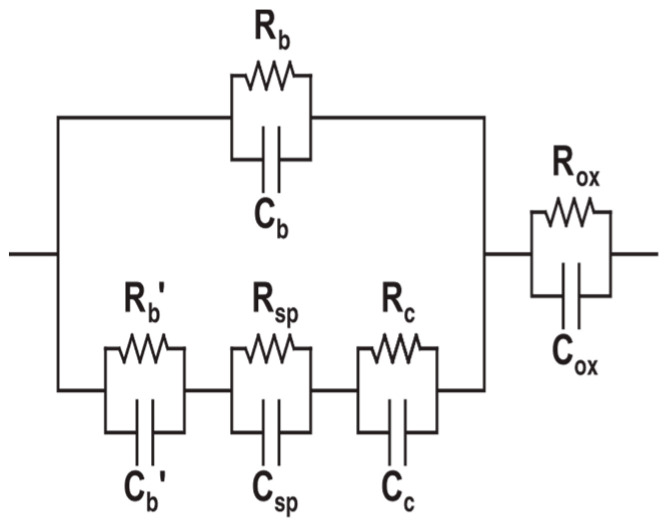	R_ox_ is the resistance due to the oxide film formation on the copper-mesh electrodes, and C_ox_ is the capacitance due to the electrode films or double layers. R_c_ and C_c_ are the resistance and capacitance due to the fibre oxide coating, respectively. R_sp_ and C_sp_ are the spreading resistance and capacitance at fibre tips, respectively. R_b_^/^ and C_b_^/^ are the bulk resistance and the bulk capacitance between adjacent fibres, respectively. R_b_ and C_b_ are the bulk resistance and capacitance of the matrix, respectively.	The model was used to study the non-linear relationship between current and voltage in cement-based composites containing steel fibres.	The threshold of the four-point DC resistance measurement was ±50 mA, with a range of frequencies of 11–100 MHz. Additionally, measuring the resistance for a long period of time leads to the corrosion of fibre tips.	[174]
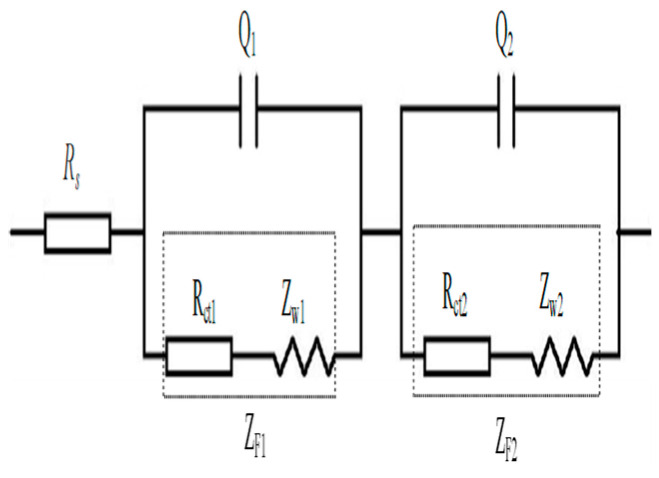	R_s_ simulates the resistance of electrolyte solutions. Q_1_ represents the double-layer capacitance on the surface of multi-walled CNTs, R_ct1_ simulates the resistance caused by charge transfer on the surface of multi-walled CNTs, and Z_w1_ simulates the Warburg resistance due to charge diffusion on the surface of multi-walled CNTs. Q_2_ represents the double-layer capacitance between cement material and electrodes, R_ct2_ simulates the resistance due to charge transfer on the surface of electrodes, and Z_w2_ represents the Warburg resistance due to charge diffusion on the surface of electrodes.	The model was used to study the fracture toughness of multi-walled carbon nanotube/cement composites.	The model was limited to carbon nanotubes with lengths and diameters of 10–30 µm and 10–20 nm, respectively. The CNT was up to 0.1 wt%.	[175]
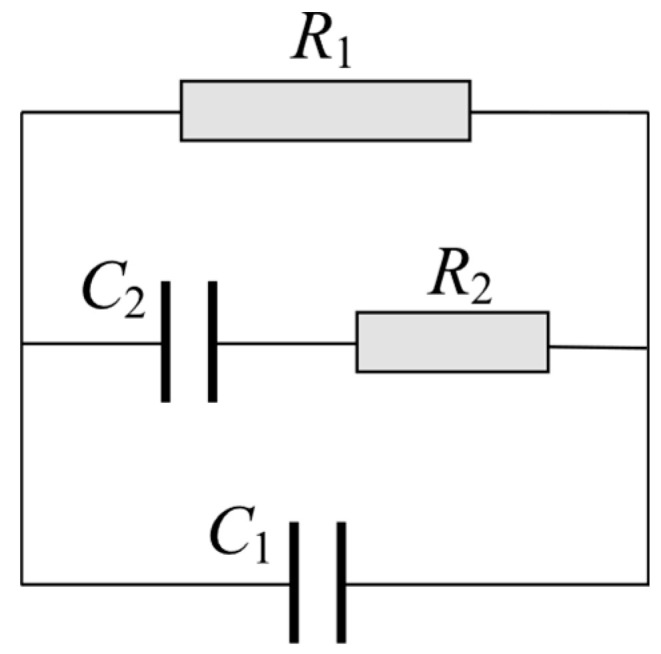	R_1_ simulates the bulk resistance of conductive paths, and R_2_ represents the resistance of partially conductive paths. C_1_ simulates the capacitance of non-conductive paths, and C_2_ represents the capacitance of partially conductive paths.	The model was used to study the electrical properties of cement-based composites containing carbon black nanoparticles and PVA fibres.	The model was used to simulate the high-frequency region, excluding the electrode/sample interface. It was restricted to uncracked bulk samples containing PVA fibres at 2% by volume and CB up to 10%.	[176]
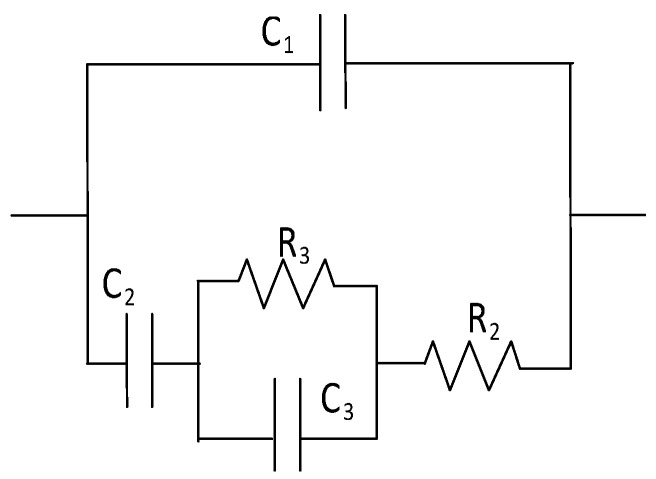	R_2_ is a resistor representing the electrolyte filling the non-percolating pores, and R_3_ is a resistor simulating the charge transfer across the fibre/electrolyte interface. C_1_ is a capacitor representing the solid phase, C_2_ is a capacitor simulating the electrolyte filling the non-percolating pores, and C_3_ is a double-layer capacitor simulating the fibre/electrolyte interface.	The model was used to study the microstructure of cementitious materials incorporating short carbon fibres.	The model was used to simulate the high-frequency region, excluding the electrode/sample interface. It was restricted to cement-based samples containing carbon fibres with a length of 5.5 mm and up to 1% by weight of cement.	[177]
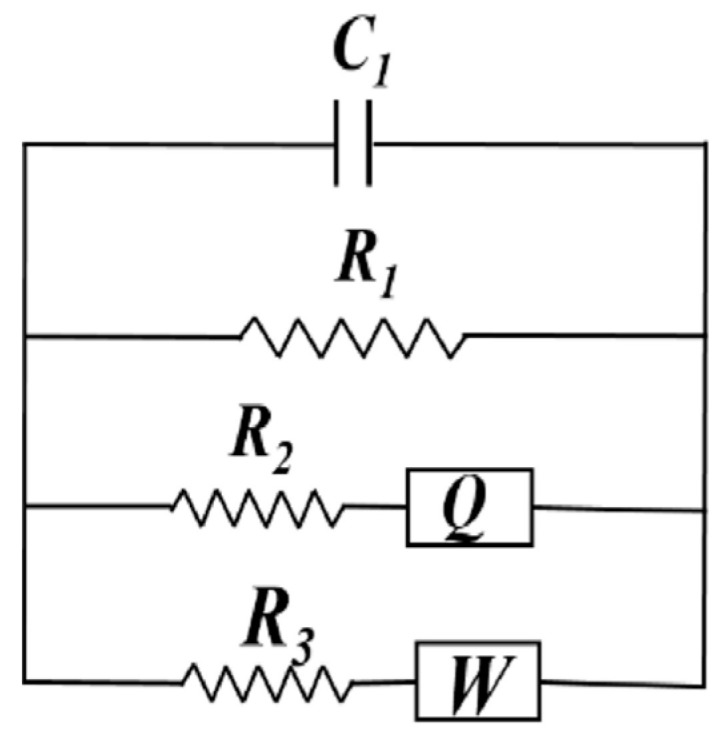	C_1_ is a capacitor representing the insulating matrix, and R_1_ is a resistor simulating carbon fibre networks or connected solutions. R_2_/Z_Q_ is a resistor in addition to a constant phase element to simulate the complex unconnected pore structure, and R_3_/Z_w_ is a resistor in addition to the Warburg element to simulate the diffusion.	The model was used to study the conductive mechanisms of cementitious materials incorporating short carbon and PVA fibres.	The model was restricted to cement-based samples containing carbon fibres with a length of 9 mm and up to 3% by weight of cement.	[178]
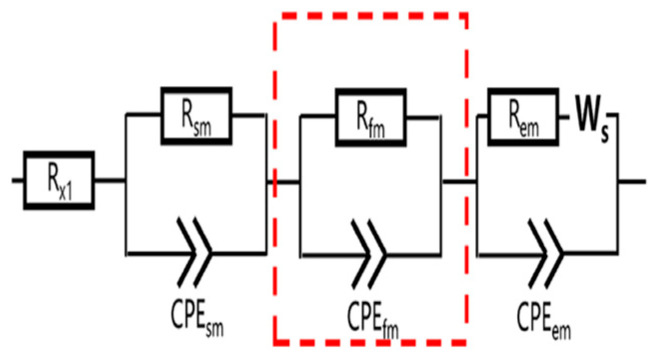	R_x1_ is a resistor representing the left intercept of the arc and the real axis. R_sm_/CPE_sm_ and R_fm_/CPE_fm_ simulate the responses from the steel fibre/matrix interface and the few-layer graphene/matrix interface, respectively. The R_em_/CPE_em_/W_s_ circuit simulates the response from the electrode/matrix interface.	The model was used to study the piezoresistive behaviour of smart ultra-high-performance fibre-reinforced concrete incorporating few-layer graphene nanomaterials as a conductive filler.	The cementitious matrix was composed of cement, quartz powder, and quartz sand with proportions of 743, 250, and 1070 kg/m^3^, respectively.	[179]
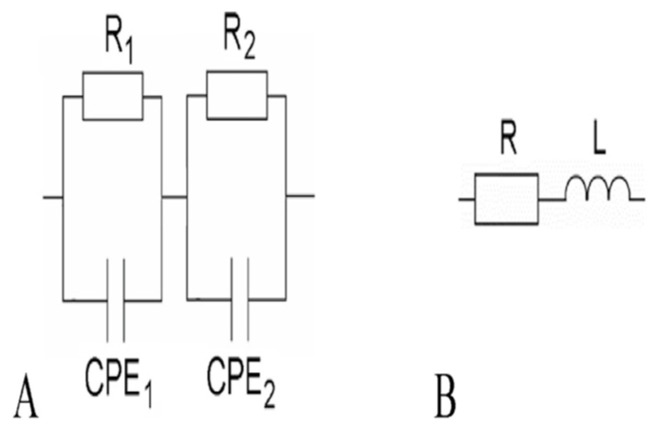	Model A represents cement-based composites containing a low percentage of expanded graphite. This model contains two loops: loop one includes a resistor (R_1_) and a constant phase element (CPE_1_) to simulate the cement/graphite interface in the high-frequency region; loop two incorporates a resistor (R_2_) and a constant phase element (CPE_2_) to simulate the electrode/matrix interface in the low-frequency region. On the other hand, model B represents cement-based composites containing a high percentage of expanded graphite. It comprises a resistor (R) and an inductor (L) in a series circuit.	The model was used to study the percolation threshold of cement-based composites containing expanded graphite.	The model was restricted to intercalated graphite type EG 290 as the conductive medium, with bulk densities of 0.016 and 0.04 gm/cm^3^ at 500 and 1000 degrees Celsius, respectively. The percolation threshold obtained from IS was lower than DC measurements.	[180]
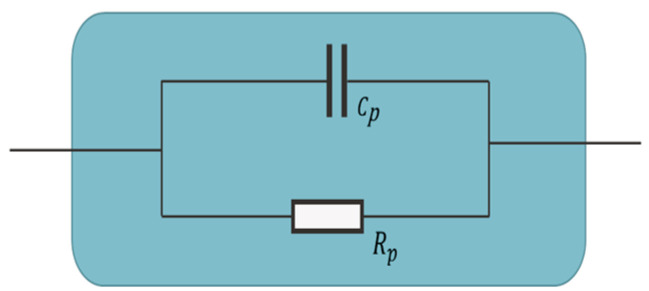	The model includes a resistor (R_p_) to simulate the bulk resistance of the cement-based sensor between electrodes and a capacitor (C_p_) to simulate the polarisation processes in the same sample.	The model was used to study the electrical properties of smart ultra-high-performance concrete containing steel fibres as conductive fillers.	The model was for the high-frequency region, excluding the electrode/sample interface. It was restricted to a cementitious matrix containing copper-coated steel fibres with a length of 13 mm, a diameter of 0.22 mm and up to 2% by volume.	[181]

## Data Availability

Not applicable.
